# Flash Glucose Monitoring and Patient Satisfaction: A Meta-Review of Systematic Reviews

**DOI:** 10.3390/ijerph18063123

**Published:** 2021-03-18

**Authors:** Ana Díez-Fernández, María Dolores Rodríguez-Huerta, Rubén Mirón-González, José Alberto Laredo-Aguilera, Noelia María Martín-Espinosa

**Affiliations:** 1 Centro de Estudios Sociosanitarios, Universidad de Castilla-La Mancha, 16071 Cuenca, Spain; ana.diez@uclm.es; 2 Facultad de Enfermería de Cuenca, Universidad de Castilla-La Mancha, 16071 Cuenca, Spain; 3 Departamento de Enfermería, Fisioterapia y Terapia ocupacional, Universidad de Castilla-La Mancha, Campus Universitario s/n, 02071 Albacete, Spain; JoseAlberto.Laredo@uclm.es; 4 Intensive Care Unit, La princesa University Hospital, 28006 Madrid, Spain; lolaorkjo@gmail.com; 5 Community Care and Social Determinants of Health Research Group, Department of Nursing and Physiotherapy, University of Alcalá de Henares, 28805 Alcalá de Henares, Spain; noelia.martin@uclm.es; 6 Grupo de Investigación Multidisciplinar en Cuidados (IMCU), Campus de Fábrica de Armas, Universidad de Castilla-La Mancha, Av de Carlos III, nº 21, 45071 Toledo, Spain; 7 Facultad de Fisioterapia y Enfermería, Campus Fábrica de Armas, Universidad de Castilla-La Mancha, Av de Carlos III, nº 21, 45071 Toledo, Spain

**Keywords:** flash glucose monitoring, quality of life, patients’ satisfaction, diabetes mellitus, meta-review

## Abstract

Flash glucose monitoring (FGM) systems have been suggested to have clinical beneficial effects in patients with diabetes mellitus, although their improvements in terms of quality of life (QoL) and patients’ satisfaction are not always addressed or are considered a secondary outcome. Thus, the aim of this meta-review is to establish the benefits of FGM in terms of patients’ satisfaction and QoL in both type 1 and type 2 diabetes patients using evidence from past systematic reviews and meta-analyses. Major databases were searched for systematic reviews (with or without meta-analyses) that assessed the satisfaction or QoL of type 1 or 2 diabetes patients using FGM compared with other glucose monitoring systems. The quality of the included systematic reviews was addressed with the Assessment of Multiple Systematic Reviews 2 (AMSTAR-2) tool. Six systematic reviews (including two meta-analyses) were included in the meta-review. Evidence suggests that FGM systems seem to improve patients’ satisfaction and QoL compared with self-monitoring of blood glucose, although the high variability in the measurement tools, the clinical significance and the quality of the systematic reviews included do not allow us to state FGM benefits with any certainty. Further research, including high-quality randomised clinical trials, differentiating the needs of both type 1 and type 2 diabetes patients and focusing on psychosocial benefits for these patients is needed to optimise clinical decisions between patients and professionals by developing the right health technology assessment for FGM systems.

## 1. Introduction

Adequate glycaemic control during the lifespan has always been a challenge for patients with both type 1 (T1D) and type 2 diabetes (T2D) and their caregivers [1]. Taking into account the need to individualise the control and goals of these patients, along with the importance of providing them with educational and technological resources, there has recently been an important evolution of treatments and self-management tools [1,2]. Specifically, the monitoring of blood glucose has evolved greatly in the last two decades, when self-monitoring of blood glucose (SMBG) ceased to be the only outpatient form of glucose control [3].

Continuous glucose monitoring provided patients and caregivers with a “full picture”, with a demonstrated improvement in glycaemic control in patients with elevated glycated haemoglobin (HbA_1c_) and an important reduction of episodes of hypoglycaemia in high-risk patients [4]. However, this system is not exempt from some impediments, such as the device’s cost, duration or need for calibration [2,5].

The emergence of a flash glucose monitoring system (FGM) in 2014 once again changed the basis of patient glycaemic control. This system, by measuring glucose at the interstitial level, allows for intermittent but fast and sufficiently accurate glucose monitoring [6,7]. It was created to respond to the limitations of SMBG and several factors of continuous glucose monitoring, such as the cost, user acceptability or accuracy of earlier devices [6].

FGM makes it possible to instantly measure interstitial blood glucose using a sensor that can be worn for 14 days and a device, such as a reader or a mobile phone, without the need for calibration. In contrast with continuous glucose monitoring, glucose values and trends can only be viewed after scanning the sensor with the FGM system, but the ubiquity of this device in a relatively short time and its adequate accuracy and simplicity have supported its acceptance by both patients and healthcare professionals [8].

Patients’ satisfaction, together with confidence in their glucose control system, is related to increased quality of life (QoL) [9,10,11], which has been addressed in different studies, systematic reviews and meta-analyses, generally as a secondary outcome.

Thus, it is interesting to address and explore the available evidence regarding the satisfaction and QoL of patients wearing FGMs, the newest system for glucose control. The aim of this meta-review was to establish the benefits of FGM in terms of satisfaction and QoL in both T1D and T2D patients using evidence from past systematic reviews and meta-analyses.

## 2. Materials and Methods

This meta-review follows the guidelines of the Preferred Reporting Items for Systematic Reviews and Meta-Analyses (PRISMA) [12]. The protocol was previously registered in the International Prospective Register of Systematic Reviews (PROSPERO) (registration number: CRD42020211979).

### 2.1. Search Strategy

Two independent researchers conducted searches in five electronic databases (MEDLINE (via PubMed), EMBASE, Web of Science, Scopus and the Cochrane Database of Systematic Reviews) to identify peer-reviewed systematic reviews of relevance written in English, French, Portuguese or Spanish that were published between January 2014 and November 2020.

Search terms included were (“Flash Glucose Monitoring” OR “freestyle libre” OR “intermittent-scanned continuous glucose monitoring”) AND (“Continuous glucose monitoring” OR “finger-stick test”) AND (“Patient Satisfaction” OR “Quality of life” OR “health-related quality of life” OR “HRQoL” OR “QoL”) AND (T2D OR “type 2 diabetes” OR “type 2 diabetes mellitus” OR “Diabetes mellitus, Type 2” OR T1D OR “type 1 diabetes” OR “type 1 diabetes mellitus” OR “Diabetes mellitus, Type 1” AND (“systematic review” OR “review” OR “meta-analysis”) (Table 1).

### 2.2. Selection Criteria

The criteria for including studies were as follows: (i) systematic reviews with or without meta-analysis, (ii) assessing patients’ satisfaction or QoL of patients using FGM, (iii) including T1D or T2D patients, (iv) comparison with continuous glucose monitoring systems or SMBG or without comparison. There was no restriction of age or race. Exclusion criteria were bibliographic reviews without a search strategy or clearly described inclusion/exclusion criteria or those not written in English, Spanish, French or Portuguese. The references selected for inclusion were entered into Mendeley Reference Management software, and duplicates were removed. Two independent researchers (M.D.R.-H. and N.M.M.-E.) selected the articles independently and in parallel according to inclusion and exclusion criteria, and a third researcher (A.D.F.) reviewed the selection in case of disagreement.

### 2.3. Data Extraction and Data Synthesis

Two researchers (R.M.-G. and J.A.L.-A.) performed the data extraction by using a form specifically designed for this purpose which summarised the following data: first author, publication year, search database, search period, design and number of all studies included, sample characteristics and type of diabetes, intervention, comparator, number and design of studies including patient satisfaction or QoL results, and inclusion or not of meta-analysis. A third researcher (A.D.F.) compared both forms and presented the final data collection.

### 2.4. Quality Assessment

The quality of the systematic reviews included was assessed by two reviewers (R.M.-G. and J.A.L.-A.) using the Assessment of Multiple Systematic Reviews (AMSTAR) 2 measurement tool, developed specifically to assess the quality of systematic reviews with reference to the methodological and systematic rigour and synthesis of the evidence [13]. This tool includes 16 items to rate the overall confidence in the results of the review and propose a scheme to interpret weaknesses in different items (from high to critically low). Any disagreements between both reviewers were resolved first by verifying the protocol criteria by consensus.

## 3. Results

### 3.1. Study Characteristics

**Figure 1:**
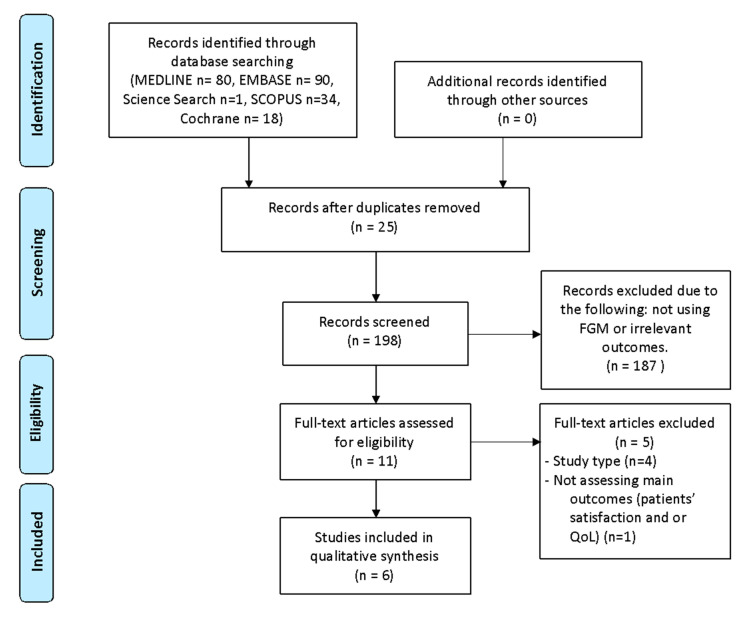
Literature search Preferred Reporting Items for Systematic Reviews and Meta-Analyses (PRISMA) consort diagram.

The literature search retrieved 198 records, which were screened by reading titles and abstracts, of which 11 were identified for full-text inspection. After reading them carefully, five articles were removed because they did not meet the inclusion criteria. Finally, six systematic reviews were included in this analysis (Figure 1).

These systematic reviews included in this meta-review (Table 2) cited seven randomised controlled trials (RCTs) [14,15,16,17,18,19], four observational studies [20,21,22,23,24] and two letters [25,26]. They all analysed the outcomes of patients’ satisfaction and/or QoL with the use of FGM vs. SMBG in T1D and/or T2D.

Two of the included systematic reviews with high methodological quality [27,28] constitute extensive evaluations of the technology, evaluating effectiveness, safety and cost-effectiveness mainly, and QoL and patient satisfaction as main or secondary important objectives. Additionally, one is a network meta-analysis that evaluates all possible technologies and combinations of insulin measurement and delivery, with a small portion focused on FGM [28]. Finally, we included four systematic reviews considered to be of moderate or low quality (Table 3).

### 3.2. Quality of Life

Different scales were used to evaluate this item:

Diabetes Quality of Life (DQoL) questionnaire: DQoL scores range from 1 to 5; high scores indicate dissatisfaction, frequent impact or frequent worry [30].

Diabetes Distress Scale (DDS): a 17-item scale that captures four critical dimensions of distress: emotional burden, regimen distress, interpersonal distress and physician distress. Each item is rated on a six-point scale from “not a problem” to “a very significant problem [31].

The Five-Item World Health Organization Well-Being Index (WHO-5) consists of five statements, which respondents rate from “All of the time” to “At no time”. The total raw score, ranging from 0 to 25, is multiplied by 4 to give the final score, with 0 representing the worst imaginable well-being and 100 representing the best imaginable well-being [32].

PedsQL™ in Type 1 and Type 2 Diabetes consists of 23 items evaluating (1) physical functioning (8 items), (2) emotional functioning (5 items), (3) social functioning (5 items), and (4) school functioning (5 items). The instructions ask how much of a problem each item has been during the past month. A five-point response scale is used, from “never a problem” to “almost always a problem” [33].

The Audit of Diabetes-Dependent Quality-of-Life (ADDQoL) assesses the impact of diabetes on 19 life domains: physical functioning, symptoms, psychological well-being, social well-being, role activities and personal constructs [34].

The review of Ang et al. (2020) shows that the RCTs of Bolinder et al. [14] and Yaron et al. [16] did not show a significant improvement in DQoL and ADDQoL scores in T1D and T2D patients, respectively. This review also reported DDS and WHO-5 scores, both statistically significant in favour of FGM (*p* = 0.006 and *p* < 0.001, respectively) (Table 4). Additionally, this review is the only one that cited a qualitative analysis in T1D patients [22] that discussed the QoL when using FGM. The users of the technology argued an improvement in their QoL, and their family or caregivers improved their empowerment and confidence in managing the disease.

The review of Bidonde et al. (2017), considered of moderate quality, and one of the two included that performed a meta-analysis, included one RCT for T1D [14] and one RCT for T2D [15]. Their results showed no significant differences in QoL (using the DQoL scale) between FGM and SMBG, with a mean difference of −0.05 (95% CI: −0.16 to 0.05), with low heterogeneity (Table 4).

Of the nine RCTs included in the review by Cowart et al. (2020), the results of QoL are shown in only one study, the one by Haak et al. [15], reporting a significant improvement in DQoL score in the FGM group: −0.2 ± 0.4 vs. 0.0 ± 0.06 (Table 4). It is interesting to address that, despite including the study by Bolinder et al. [14] in this review, this QoL variable was not included in the results.

In the review of Dicembrini et al. (2019), results concerning QoL were not quantitatively specified, but they included the study by Haak et al. in T2D patients, which showed an improvement in QoL with the use of FGM vs. SMBG [15] (Table 4).

The review published by a multidisciplinary team from Health Quality Ontario in 2019, in a series to assess technology in diabetes, included QoL and patients’ preferences in their results, reporting statistically significant improvements in QoL in T1D patients using FGM vs. SMBG [20,23]. Nevertheless, these differences cannot be considered clinically significant. This review, considered of high quality, also included an RCT with a statistically nonsignificant difference between FGM and SMBG (mean difference of −0.08; 95% CI: −0.16 to 0.00) [14]. Studies that evaluated T2D patients did not observe any differences between groups [15,20] (Table 4). The authors also report that adult patients and parents of children with diabetes positively valued the FGM system, since they believed it helped to improve glycaemic control and brought physical, social and emotional benefits.

According to the network meta-analysis of Pease et al. (2020), with 52 studies included, the combination of multiple doses of insulin (MDI) with FGM is the second-best treatment option in terms of QoL (surface under the cumulative ranking curve (SUCRA) 66.3%), after MDI with CGM (SUCRA 88.9%). The review included two studies in T1D patients using the FGM system, referring to nonsignificant differences among groups in DQoL [14] scores and the DDS [14,19] (Table 4).

### 3.3. Patients’ Satisfaction

The Diabetes Treatment Satisfaction Questionnaire (DTSQ) was used to evaluate this item. It includes eight questions, scored from zero (e.g., “very dissatisfied”, “very inconvenient”) to six (e.g., “very satisfied”, “very convenient”). The second factor assesses the burden of hyper- and hypoglycaemia (from “none of the time” to “most of the time”) in two questions. Treatment satisfaction is assessed as the sum of the scores of the six questions on the first factor (total score: 36), with a higher score indicating higher treatment satisfaction [35].

According to the review of Ang et al. (2020), Yaron et al. indicated that 87.5% of the group who used FGM were very satisfied and would recommend FGM to the control group [16]. All the included studies with results from the DTSQ showed better results with the FGM system [14,16,21,22] (Table 4).

According to the meta-analysis of Bidonde et al. (2017), FGM may improve treatment satisfaction for individuals with T1D or T2D, but the quality of this evidence was low due to substantial clinical and statistical heterogeneity. Despite high heterogeneity, both groups separately improved their treatment satisfaction at the end of the intervention (Table 4).

In the review of Cowart et al. (2020), two RCTs reported outcomes on T2D patients’ satisfaction, finding statistically significant improvements with the use of FGM [15,17]. In addition, according to Hermanns et al. (2019), a structured diabetes education programme combined with CGM may also improve patients’ satisfaction, since they found that the FGM group’s satisfaction was higher, but not significantly so, compared with the usual care group (−1.2, 95% CI: −2.8 to 0.3; *p* = 0.118) (Table 4).

Lastly, the review of Pease et al. (2020), which included DTSQ results reported by Oskarsson et al. [19], favoured FGM over SMBG, albeit without reporting statistical significance values (Table 4).

## 4. Discussion

As far as we know, this current meta-review is the first to synthesise and analyse an overview of the benefits of using FGM in terms of satisfaction and QoL in both T1D and T2D patients. Our meta-review shows that the use of FGM in this population generally improves their QoL, but we should interpret these results with caution due to the high heterogeneity in the design and quality of the primary studies included in the six systematic reviews analysed, which also used different tools and questionnaires to assess these variables.

It has been described that QoL in patients with diabetes may be associated with several sociodemographic factors and its improvement is usually linked to adequate glycaemic control in both T1D and T2D patients [9]. In particular, QoL in T1D patients can be compared with healthy peers, with the exception of disease-specific QoL problems, such as diabetes-related worries, or a negative impact of diabetes on daily functioning [36]. Regarding T2D patients, a systematic review and meta-analysis concluded that the appearance of complications is importantly related to a lower QoL [11].

In the present meta-review, the two reviews classified as high-quality did not report statistically significant differences in QoL between the group using FGM and the different control groups, and when those differences were found, they were irrelevant from a clinical perspective [27,28]. One of these reviews, in line with our results, pointed out that there are some limitations, with some inconsistency of results and potential reporting bias, so they rated the evidence for QoL as very low in patients with diabetes using FGM in comparison with other devices [27]. Despite this, the authors highlighted that FGM seemed to have a positive influence on patients’ QoL.

Regarding patients’ satisfaction, only four of the systematic reviews included assessed this variable [28,37,38,39], concluding that patients using FGM seemed to report a higher satisfaction than those using other glucose measuring tools. Nevertheless, some studies showed incomplete results for this variable, and the information reported does not allow us to evaluate the methods used to measure it. Additionally, the scientific evidence can be considered low quality due to the studies’ heterogeneity and the poor methodological quality of the reviews.

Despite the good acceptance of the FGM system, the novelty of this device and its cost in countries where this device is not financed have made its use unlikely for the entire population with diabetes. This could be the reason for the scarcity of studies focused on the use of this technique. Additionally, some device-related adverse events have been described, such as erythema, rash, itching, pain, bleeding, oedema, induration or bruising [27,37,38]. In this regard, no evidence is available on whether these effects may negatively affect QoL or patients’ satisfaction. Subjective aspects related to FGM such as patients’ QoL and satisfaction have been much less studied than others associated with physiological aspects of the disease such as glycaemic control or HbA1c levels.

Furthermore, the second generation of FGM was launched in 2018, and it provides optional alarms for trends toward high or low glucose levels on a patient’s mobile phone, allowing the patient to scan the sensor and verify the situation, which should lead to an improvement in the acceptability and use of the device [40]. Further research on this evolved FGM system is needed to address whether patients’ QoL and satisfaction with their daily life from an objective and subjective standpoint increase from the previous version. Whereas its inclusion with hybrid closed-loop systems has been reported in T1D patients [41,42], its use is apparently unsuitable for the development of the artificial pancreas [43], which may be an important factor in considering it as a glucose measurement system with these hybrid insulin administration models in the future.

Finally, it is important to consider that the way of perceiving and coping with diabetes could be different between different age ranges. Thus, more studies in the paediatric population should be conducted [43] to determine the long-term impact of using FGM on physiological and psychological aspects in adolescents and young adults [6]. Considering that the use of FGM is not allowed in people younger than 18 years in some countries [7], more research in this specific age group may help to modify this policy if its use results are shown to be beneficial, as different studies have reported on the families of children with diabetes [29,44].

Despite these promising findings, it is important to consider some limitations of the present meta-review. Firstly, the great heterogeneity observed in the methodological quality of the systematic reviews included and the mixed quality of the studies included in the latter reward us for interpreting the findings with caution. Perhaps this is the reason for the scarcity of meta-analyses on the studied variables. Thus, high-quality research is necessary to confirm our results. Secondly, the primary studies have used different tools for evaluating patients’ QoL and satisfaction, and their description is not always accurately explained, so a comparison between the findings of different studies assessing the variables studied should be made with caution as well.

Thirdly, although the use of FGM seems to improve the QoL and satisfaction of both T1D and T2D patients, it would be appropriate to deeply analyse these variables in each type of diabetes separately and to qualitatively analyse which are the needs and worries of FGM users. Despite the fact that Rodriguez-Almagro et al. concluded that health-related QoL in people with diabetes is not conditioned by the type of diabetes [9], it is well known that the pathological condition of both populations is different, and this might lead to different conclusions regarding the integration of this technology in the development of their respective future treatments.

## 5. Conclusions

The emergence of FGM seems to have changed the way diabetes care is managed. However, the repercussions of its use in patients’ daily life in terms of QoL and satisfaction have scarcely been studied, although the published evidence suggests that FGM could improve these aspects. Further RCTs in different age groups, differentiating T1D and T2D, and evaluating differences with other monitoring systems, such as CGM, should be designed to assess its effects on subjective aspects affecting individuals with diabetes. These types of studies could be useful to optimise clinical decisions between patients and professionals by developing the right health technology assessment for FGM systems.

## Figures and Tables

**Table 1:** Search strategy for MEDLINE.

1	Finger-stick test	14	T2D
2	Continuous glucose monitoring	15	type 2 diabetes mellitus
3	#1 OR #2	16	Diabetes mellitus, Type 2
4	Flash glucose monitoring	17	T1D
5	Freestyle Libre	18	type 1 diabetes
6	Intermittent-scanned continuous glucose monitoring	19	type 1 diabetes mellitus
7	#4 OR #5 OR #6	20	Diabetes mellitus, Type 1
8	Quality of life	21	#14 OR #15 OR #16 OR #17 OR #18 OR #19 OR #20
9	Patient Satisfaction	22	systematic review
10	Health-related quality of life	23	review
11	HRQoL	24	meta-analysis
12	QoL	25	#22 OR #23 OR #24
13	#8 OR #9 OR #10 OR #11 OR #12	26	3 AND 7 AND 13 AND 21 AND 25

Abbreviations: HRQoL: health-related quality of life; QoL: quality of life; T2D: type 2 diabetes mellitus; T1D: type 1 diabetes mellitus.

**Table 2:** Characteristics of the included systematic reviews.

First Author (Year)	Search Databases and Search Period	Design of Included Studies, n	Total Sample, n Intervention/Comparator	Patients’ Satisfaction and QoL Design of Included Studies, n	First Author of Included Studies in Each Systematic Review with QoL and/or Patients’ Satisfaction Results	Meta-Analysis
Ang (2020)	MEDLINE, EMBASE, period not reported	RCTs PC RP Letters *n* = 16	1901 T1D and T2D adult patients. FCG/CGM or not specified	RCTs, *n* = 2 PC, *n* = 3 RP, *n* = 1 Letter, *n* = 2	Bolinder [14], Yaron [16], Mitsuishi [20], Kramer [21], Nana [24], Overend [22], Ish-Shalom [25], Dover [26]	No
Bidonde (2017)	MEDLINE, Embase, Cochrane Library, Centre for Reviews and Dissemination: Database of Abstracts of Reviews of Effects, Health Technology Assessment database and other sources up to 18 January 2017	RCTs *n* = 2 and their protocols	465 T1D and T2D adult patients. FCG/SMBG	RCTs, *n* = 2	Bolinder [14], Haak [15]	Yes
Cowart (2020)	Embase, PubMed, and the Cochrane Library CENTRAL Register of Controlled Trials, from each index’s inception through 8 November 2019	RCTs *n* = 9	689 all T1D and T2D children, adolescents, adults and gestational diabetes. FCG/SMBG	RCTs, *n* = 4	Haak [15], Ajjan [17], Yaron [16], Hermanns [18]	No
Dicembrini (2019)	MEDLINE up to 1 September 2018	RCTs *n* = 12	224 T2D adult patients. FCG/SMBG	RCTs, *n* = 1	Haak [15]	No
Ontario Health (2019)	MEDLINE, Embase, the Cochrane Central Register of Controlled Trials, the Cochrane Database of Systematic Reviews, the Health Technology Assessment Database, and the National Health Service Economic Evaluation Database up to April, 2018	RCTs Observational studies *n* = 6	918 T1D and T2D with no restriction of age. FCG/SMBG	RCTs = 1 Observational *n* = 2	Al Hayek [29], Bolinder [14], Mitsuishi [20]	No
Pease (2020)	MEDLINE, MEDLINE In-Process, EMBASE, PubMed, All Evidence-Based Medicine Reviews, Web of Science, PsycINFO, CINAHL PROSPERO (inception–24 April 2019)	RCTs *n* = 52	3975 T1D adults. Comparison among CGM, SMBG and FGM.	RCTs, *n* = 2	Bolinder [14], Oskarsson [19]	Yes

Abbreviations: RCT: randomised clinical trial; PC: prospective comparative study; RP: retrospective cohort; T1D: type 1 diabetes mellitus; T2D: type 2 diabetes mellitus; QoL: quality of life; FGM: flash glucose monitoring; SMBG: self-monitoring blood glucose; CGM: continuous glucose monitoring.

**Table 3:** Assessment of Multiple Systematic Reviews 2 (AMSTAR-2) ratings of systematic reviews and meta-analyses.

	1	2	3	4	5	6	7	8	9	10	11	12	13	14	15	16	
**Ang et al. 2020**	Yes	No	Yes	Partial Yes	Yes	Yes	No	Partial Yes	No	No	No MA	No MA	No	No	No	Yes	**Low**
**Bidonde et al. 2017**	Yes	No	Yes	Yes	Yes	Yes	Yes	Yes	Yes	No	Yes	Yes	Yes	Yes	Yes	No	**Moderate**
**Cowart et al. 2020**	Yes	No	Yes	Partial Yes	Yes	No	No	Yes	Yes	Yes	No MA	No MA	Yes	No	No	Yes	**Critically low**
**Dicembrini et al. 2019**	Yes	Yes	Yes	Partial Yes	Yes	Yes	No	Partial Yes	Yes	No	Yes	Yes	Yes	Yes	Yes	Yes	**Low**
**Ontario Health 2019**	Yes	Yes	Yes	Yes	Yes	Yes	No	Partial Yes	Yes	No	No MA	No MA	Yes	Yes	No	Yes	**High**
**Pease el at 2020**	Yes	Yes	Yes	Partial Yes	Yes	Yes	Yes	Yes	Yes	Yes	Yes	Yes	Yes	Yes	Yes	Yes	**High**

Key: Item Description. 1: Did the research questions/inclusion criteria include the components of PICO? 2: Did the review contain an explicit statement that the review methods were established prior to the conduct of the review? 3: Did the review authors explain their selection of the study designs for inclusion in the review? 4: Did the review authors use a comprehensive literature search strategy? 5: Did the review authors perform study selection in duplicate? 6: Did the review authors perform data extraction in duplicate? 7: Did the review authors provide a list of excluded studies and justify the exclusions? 8: Did the review authors describe the included studies in adequate detail? 9: Did the review authors assess the risk of bias in studies that were included in the review? 10: Did the review authors report on the sources of funding for the studies included in the review? 11: If meta-analysis was performed, did the review authors use appropriate methods for statistical combination of results? 12: If meta-analysis was performed, did the review authors assess the potential impact of risk of bias in individual studies on the results of the meta-analysis? 13: Did the review authors account for risk of bias in individual studies when interpreting the results of the review? 14: Did the review authors provide a satisfactory explanation for, and discussion of, any heterogeneity observed in the results of the review? 15: If they performed quantitative synthesis, did the review authors investigate publication bias? 16: Did the review authors report any potential sources of conflict of interest, including any funding they received for conducting the review?

**Table 4:** Quantitative results showed in the included systematic reviews and meta-analysis between FGM and comparator.

First Author (Year)	T1DM				T2DM			
	QoL	*p* or 95% CI	Patients’ Satisfaction	*p* or 95% CI	QoL	*p* or 95% CI	Patient’s Satisfaction	*p* or 95% CI
Ang (2020)	(1) No significant differences in DQoL scores (1RCT) (2) Significant improvement with DDS score with FGM (1RCT and 1 letter).	*p* = 0.052 *p* 0.001 and *p* = 0.006	(1) Better DTSQ score in FGM (1RCT) (2) Better DTSQ score in FGM by 12.6 ± 5.5 points (1PC).	<0.001 NR	(1) No significant differences in ADDQoL (1RCT)	NR	(1) FGM group scored better in the DTSQ (2.47 ± 0.77 vs. 2.18 ± 0.83) (1RCT)	*p* = 0.053
	**T1DM AND T2DM TOGETHER (1PC)**							
			(1) Better DTSQ score after use of FGM.	*p* = 0.001	(1) WHO-5 scored better with FGM	*p* = <0.001		
Bidonde (2017)	(1) DQoL mean difference between groups = −0.10 (1RCT)	95% CI = −0.25 to 0.05	(1) DTSQ mean difference between groups = 6.20 (1RCT)	95% CI = 4.54 to 7.86	(1) DQoL mean difference between groups = 0.00 (1RCT)	95% CI = −0.16 to 0.16	(1) DTSQ mean difference between groups = 4.00 (1RCT)	95% CI = 2.32 to 5.68
	**T1DM AND T2DM TOGETHER (M-A including 2RCT)**							
			(1) DTSQ mean difference between groups = 5.10	I^2^ = 70% 95% CI = 2.95 to 7.26.	(1) DQoL mean difference between groups = −0.05	I^2^ 0% 95% CI = −0.16 to 0.05		
Cowart (2020)					(1) Significant improvement in DQoL score in FGM group = −0.2 ± 0.4 vs. 0.0 ± 0.06 (1RCT)	*p* = 0.025	(1) DTSQ score was better in the FGM group compared with SMBG (13.1 ± 0.50 vs. 9.0 ± 0.72) (1RCT) (2) DTSQ adjusted mean FGM vs. SMBG: 3.45 ± 1.54 vs. 3.54 ± 1.52	*p* = <0.001 *p* = 0.02
Dicembrini (2019)					(1) DQoL showed better results for FGM vs. SMBG (1RCT)	NR		
Ontario Health (2019)	(1) PedsQoL mean difference in favour of FGM vs. SMBG = 3.4 (1.31–5.49) (1PC). (2) DQoL mean difference between FGM and SMBG = −0.08 (95% CI −0.16 to 0.00) (1RCT). (3) WHO-5 mean difference between FGM and SMBG = 2.1 (1PC).	*p* = 0.002 *p* = 0.052 95% CI = 0.45 to 3.75.			(1) No significant increase in QoL scores (1RCT) (2) WHO-5 mean difference between FGM and SMBG = 1.0 (1PC)	NR 95% CI = −1.16 to 3.16		
	**T1DM AND T2DM TOGETHER (1PC)**							
					(1) WHO-5 mean difference between groups (in favour of FGM) = 1.7	95% CI = 0.35 to 3.05		
Pease (2020)	(1) DQoL mean difference among groups = −0.08 (SE = 0.039) (1RCT) (2) Only the “satisfaction with treatment” subscore was significantly different and favoured FGM over SMBG (1RCT). (3) DDS mean difference −0.03 (SE = 0.089); (1RCT) and mean difference NR (1RCT). SUCRA: MDI + FGM second-best option (66.3%)	*p* = 0.052 *p* = <0.001 *p* = 0.763 and NR	DTSQ score: favoured FGM over SMBG: 13.3 (5.4) vs. 6.8 (6.2)	NR				

Abbreviations: QoL: quality of life; DQoL: Diabetes Quality of Life instrument; PedsQoL: Paediatrics Quality of Life Inventory; ADDQoL: Audit of Diabetes-Dependent Quality of Life questionnaire. WHO-5: World Health Organisation—Five Well-Being Index; DTSQ: Diabetes Treatment Satisfaction Questionnaire; FGM: flash glucose monitoring; SMBG: self-monitoring blood glucose; RCT: randomised clinical trial; PC: prospective comparative study; 95% CI: 95% confidence interval; SE: standard error; NR: not reported; MDI: multiple doses of insulin; CGM: continuous glucose monitoring; SUCRA: surface under the cumulative ranking curve.

## Data Availability

All data is available in the publication.

## Abbreviations

ADDQoL	Audit of Diabetes-Dependent Quality-of-life
AMSTAR	Assessment of Multiple Systematic Reviews
CGM	Continuous glucose monitoring
DDS	Diabetes Distress Scale
DQoL	Diabetes Quality of Life
DTSQ	Diabetes Treatment Satisfaction Questionnaire
FGM	Flash glucose monitoring
HBA_1C_	Glycated haemoglobin
HRQoL	Health-related quality of life
MDI	Multiple doses of insulin
PEDSQoL	Paediatrics Quality of Life Inventory
PRISMA	Preferred Reporting Items for Systematic Reviews and Meta-Analyses
PROSPERO	International Prospective Register of Systematic Reviews
QoL	Quality of life
RCTS	Randomised controlled trials
SMBG	Self-monitoring of blood glucose
SUCRA	Surface under the cumulative ranking curve
T1D	Type 1 diabetes
T2D	Type 2 diabetes
WHO	World Health Organization
